# Case Report: Advanced grade 2 meningioma with *PBRM1* inactivation with prolonged response to immunotherapy

**DOI:** 10.3389/fonc.2025.1587752

**Published:** 2025-10-22

**Authors:** Ellen Reusch, Keng Hee Peh, Rachael Morgan, Harry Momo, David Orren, Stephanie Rock, Thomas Pittman, Janna Neltner, Jill Kolesar, John Villano

**Affiliations:** ^1^ Markey Cancer Center, University of Kentucky, Lexington, KY, United States; ^2^ College of Pharmacy, University of Kentucky, Lexington, KY, United States; ^3^ College of Medicine, University of Kentucky, Lexington, KY, United States; ^4^ Molecular Science Liaison Group, Caris Life Sciences, Phoenix, AZ, United States; ^5^ Department of Neurosurgery, University of Kentucky, Lexington, KY, United States; ^6^ Department of Pathology and Laboratory Medicine, University of Kentucky, Lexington, KY, United States

**Keywords:** atypical meningioma, immunotherapy, PBRM1, grade 2 meningioma, PD-1/L1

## Abstract

Meningiomas are the most common primary tumor in the central nervous system, yet an effective systemic treatment remains a challenge. We present a grade 2 meningioma that resulted in a positive and prolonged response to pembrolizumab. Our case had polybromo-1 (*PBRM1*) and *BAP1* functional loss, tumor mutational burden of 4 Muts/Mb, stable microsatellite status, and a PD-L1 tumor proportion score of <1%. We add to the limited literature regarding *PBRM1* mutations in meningiomas. We discuss our findings in relation to the ongoing investigation of immune checkpoint inhibitor therapy in treating higher-grade refractory meningiomas.

## Introduction

Meningioma incidence is increasing in the US population. Meningioma is one of the rare tumors whose incidence continues to rise with advancing age (1, 2). They arise from arachnoid cells of the leptomeninges and are the most common primary tumor in the central nervous system (CNS) (1, 3). Although there are widespread asymptomatic cases in 1%–2% of the general adult population, nearly all are non-malignant grade 1 tumors (1, 4, 5). In 2016, the World Health Organization defined grade 2 meningiomas as atypical, exhibiting mitotic rates of 4–19 per 10 high power fields (HPFs), brain invasion, or at least three of five defined histological features (necrosis, sheet-like growth, prominent nucleoli, high cellularity, or high nuclear:cytoplasmic ratio within cells). Grade 3 meningiomas were considered anaplastic or malignant and were described as having mitotic rates >20 per 10 HPFs or papillary or rhabdoid histological features (6, 7). More recently, the 2021 WHO guidelines emphasize that, regardless of any underlying pathologic characteristics, atypical and anaplastic meningiomas should be classified as grade 2 and grade 3, respectively (5). Additionally, where rhabdoid and papillary features previously would be automatically classified as a grade 3 meningioma, the WHO CNS5 now recommends that meningiomas be classified based on criteria outside of those cytologic features (5). There are several molecular biomarkers that can be utilized in the classification of meningiomas. BAP1 is associated with the rhabdoid and papillary subtypes, SMARCE1 is consistent with the clear cell subtype, and KLF4/TRAF7 mutations are associated with the secretory subtype. Furthermore, meningiomas with *CDKN2A/B* homozygous deletions and/or *TERT* promoter mutations are classified as grade 3. Prognosis can be estimated through methylome profiling, and some mutations (H2K27me3 loss of nuclear expression) may be associated with poorer prognoses (5). From surgically resected cases, higher-grade meningiomas remain a minority: atypical or borderline malignant grade 2 tumors occur in 5%–15% and malignant grade 3 tumors in 1%–3% of cases (1, 4). The recurrence rates following surgery are low for grade 1 tumors but increase to 30% to 40% for grade 2 and 50% to 80% for grade 3 (1, 4).

Regarding immune access, recent anatomical discoveries demonstrate that the central nervous system is no longer considered a strictly immune-privileged organ (8). Lymphatic vessels, adjacent to the blood vascular system, are the primary means by which bodily tissues can eliminate excess fluid and proteins (9). Tissues with higher metabolic rates typically contain denser lymphatic systems. Interestingly, despite the high rate of metabolic byproduct formation, the brain and spinal cord do not contain a lymphatic tree (9, 10). Instead, waste products from the CNS are removed through the exchange of cerebrospinal fluid (CSF) and interstitial fluid (ISF) within the para-arterial interstitial space (9, 11). ISF then drains out of the CNS into the subarachnoid lymphatic-like membrane (SLYM). This recently identified structure present under the dura separates the subarachnoid space into outer and inner compartments and limits the exchange of most peptides and proteins between the two subarachnoid compartments. The recent discovery of the SLYM adds to the suggestion that CSF transport is more sophisticated than previously acknowledged (12).

We report one of the first pathologically proven cases of meningioma having a significant and prolonged response to a single agent pembrolizumab. This patient’s tumor had a truncation in the polybromo-1 (*PBRM1*) gene, which is a tumor suppressor gene involved in the control of the cell cycle, the promotion of genomic stability, and centromeric cohesion (8). Overall, *PBRM1* is mutated in nearly 40% of all clear cell renal cell carcinoma (RCC) occurrences, as well as some papillary RCCs and bladder carcinoma (13). *PBRM1* mutations are relatively uncommon in meningiomas, but when present, they are associated with papillary subtypes and often have overlapping *BAP1* mutations (14).

The occurrence of meningiomas has undergone only limited formal investigation in regard to therapies, and they currently remain among the few relatively common tumors without a Food and Drug Administration (FDA)-approved therapy. Meningiomas are chemotherapy resistant, and both targeted and immune-based therapies have been actively investigated (15–18). As discussed previously, there is evidence of an immune-based role in higher-grade meningiomas, including containing a significantly greater intra-tumoral T-cell infiltrate, inducing known local and systemic immunosuppression, a recent case of possible immune-mediated abscopal effect from radiation therapy, and several case reports of activity for immune checkpoint inhibitors (ICIs) (19, 20). With our case study, we have begun to further explore the occurrence of *PBRM1* mutations and subsequent outcomes in patients with meningiomas.

## Caris genomics study

**Figure 1:**
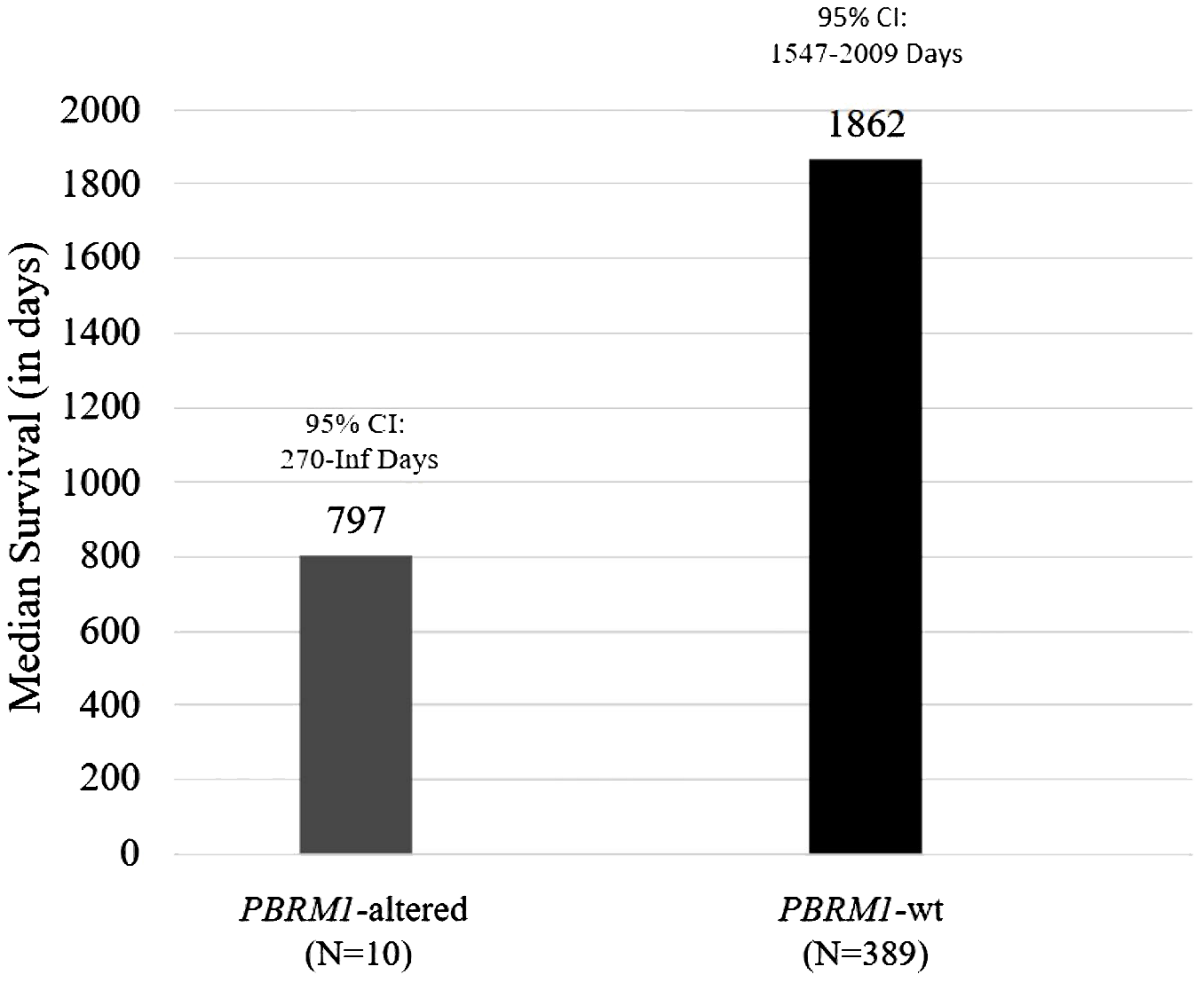
Overall survival estimates of meningioma patients with and without *PBRM1* alterations.

Genomics data from patient tumors that were sent to Caris Life Sciences for next-generation sequencing were utilized for this analysis. A total of 399 patients with meningiomas were identified, and 2.5% (n = 10) had alterations in *PBRM1*. Of the 10 patients with PBRM1 alterations, one patient had a known pathogenic point mutation variant (R1027X). Overall survival (OS) was estimated using the Kaplan–Meier method. The median OS was 797 days (95% CI 270 to ∞ days) for patients with *PBRM1* alteration and 1,862 days for those without (95% CI 1,547–2,009 days) (**Figure 1**). Of note, the patient having the *PBRM1* R1027X mutation had a survival of 797 days. The Kaplan–Meier plots and p-values were not generated due to a low sample size of *PBRM1* alterations.

## Clinical case

**Figure 2:**
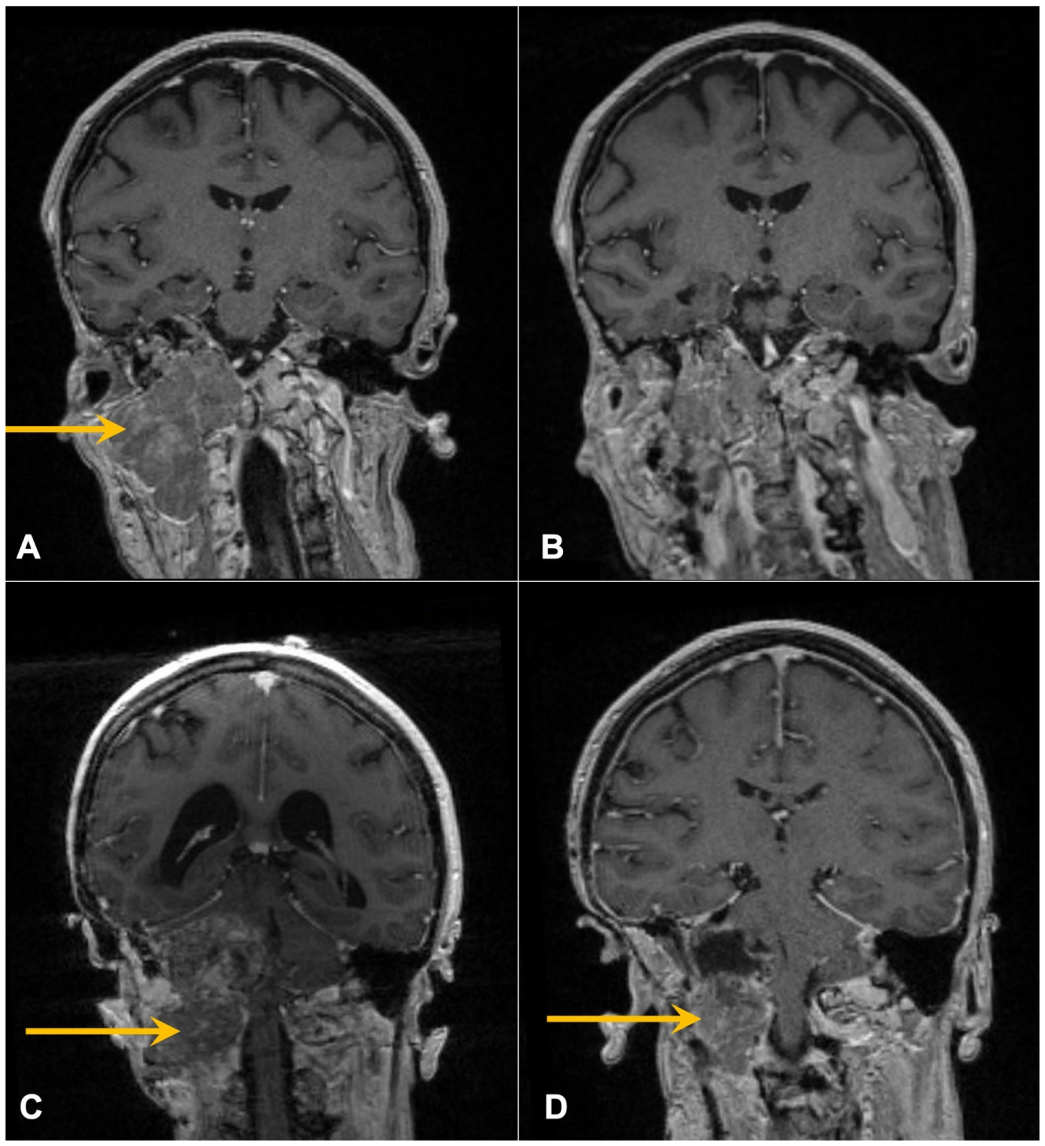
Contrast-enhanced coronal T1 MRI of the brain following recent surgeries. Arrows mark enhancing skull base mass prior to R1 **(A)** and R2 **(C)** and resulting post-operative images following R1 **(B)** and R2 **(D)**.

**Figure 3:**
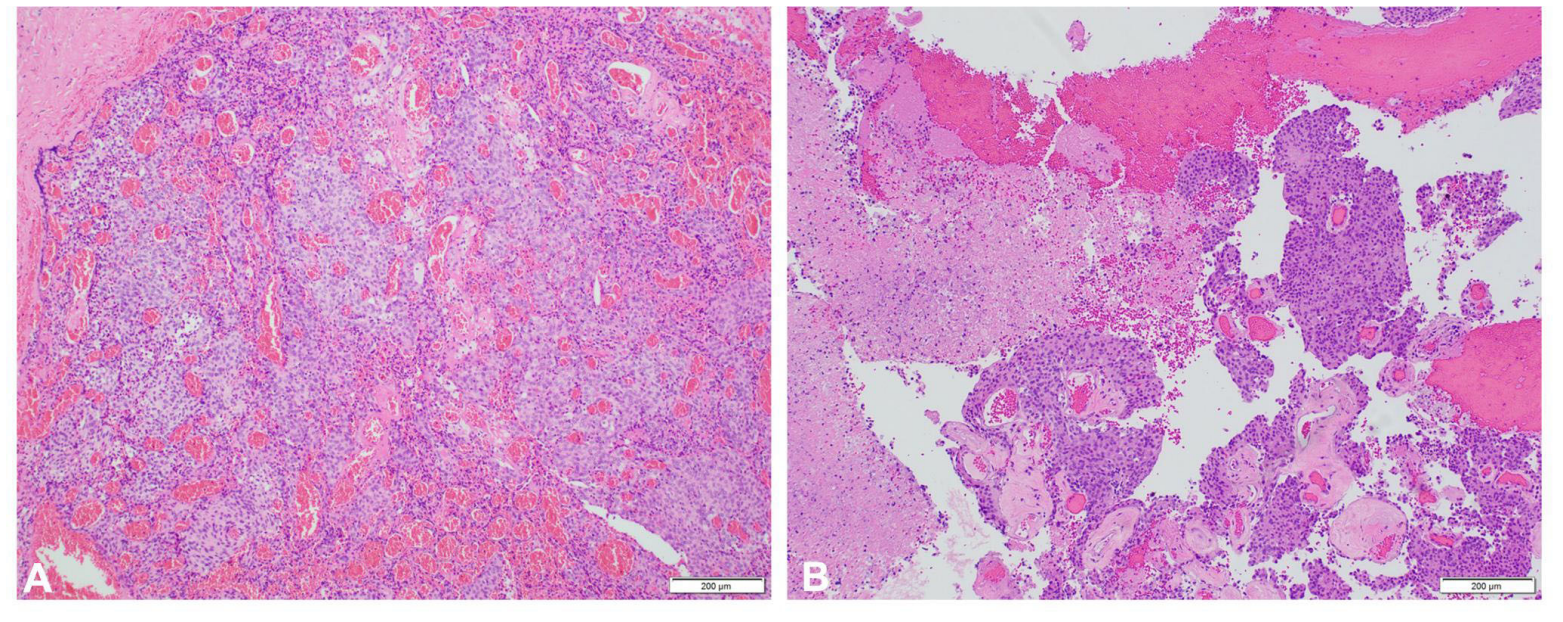
H&E, ×100 **(A, B)**. Specimens from R1 and R2 demonstrating recurrent atypical meningioma.

In 1993, at age 19, our patient was diagnosed with atypical meningioma, grade 2, located around the right mastoid region. Her treatment plan included two closely spaced surgeries and proton therapy, followed by surveillance for more than a decade. She lacked a family history of neurofibromatosis 2 (NF2) or cancer, as well as clinical or imaging evidence of NF2. Referral for genetic counseling was refused. In March 2017, at age 43, she developed progressive headaches, and imaging demonstrated an enhancing right upper neck mass with erosion of C1–C2 and extension into the posterior fossa (**Figure 2A**). Her first surgical resection (R1) following recurrence was performed in April 2017, and pathology demonstrated atypical meningioma lacking immune infiltrates. The post-surgery MRI and histopathology (H&E) images are depicted in **Figures 2B**, **3A**, respectively.

She initiated somatostatin analog injections in July 2017, which she continued monthly for 22 months until imaging in November 2018 demonstrated progression (21–23). She also developed mild headaches, tearing in her right eye, and decreased movement in the right side of her face. The decision was made to undertake a large cancer-based surgery, R2 (**Figures 2C, D**), in April 2019. Pathology from this surgery was similar to that of the R1 specimen (**Figure 3B**).

After recovery (June 2019), she enrolled in a phase I clinical trial of BXQ-350, a synthetic form of the human glycoprotein saposin C (NCT02859857). Unfortunately, she soon progressed in August 2019 with the growth of the right skull base tumor and began to have increased symptoms of headaches, dysphagia with liquids, and weight loss. To identify possible targeted therapies, next-generation sequencing (NGS) testing was performed on the R2 specimen (right cerebellar tumor) with FoundationOne. This demonstrated a non-elevated tumor mutational burden (TMB) of 4 Muts/Mb, stable microsatellite status, and no recommended therapies or trials. There were alterations including *FBXW7* G419, *BAP1* loss, and *PBRM1* loss of exons 2 to 12. The tumor was also found to have a PD-L1 tumor proportion score of <1% on PD-L1 22C3 IHC testing.

**Figure 4:**
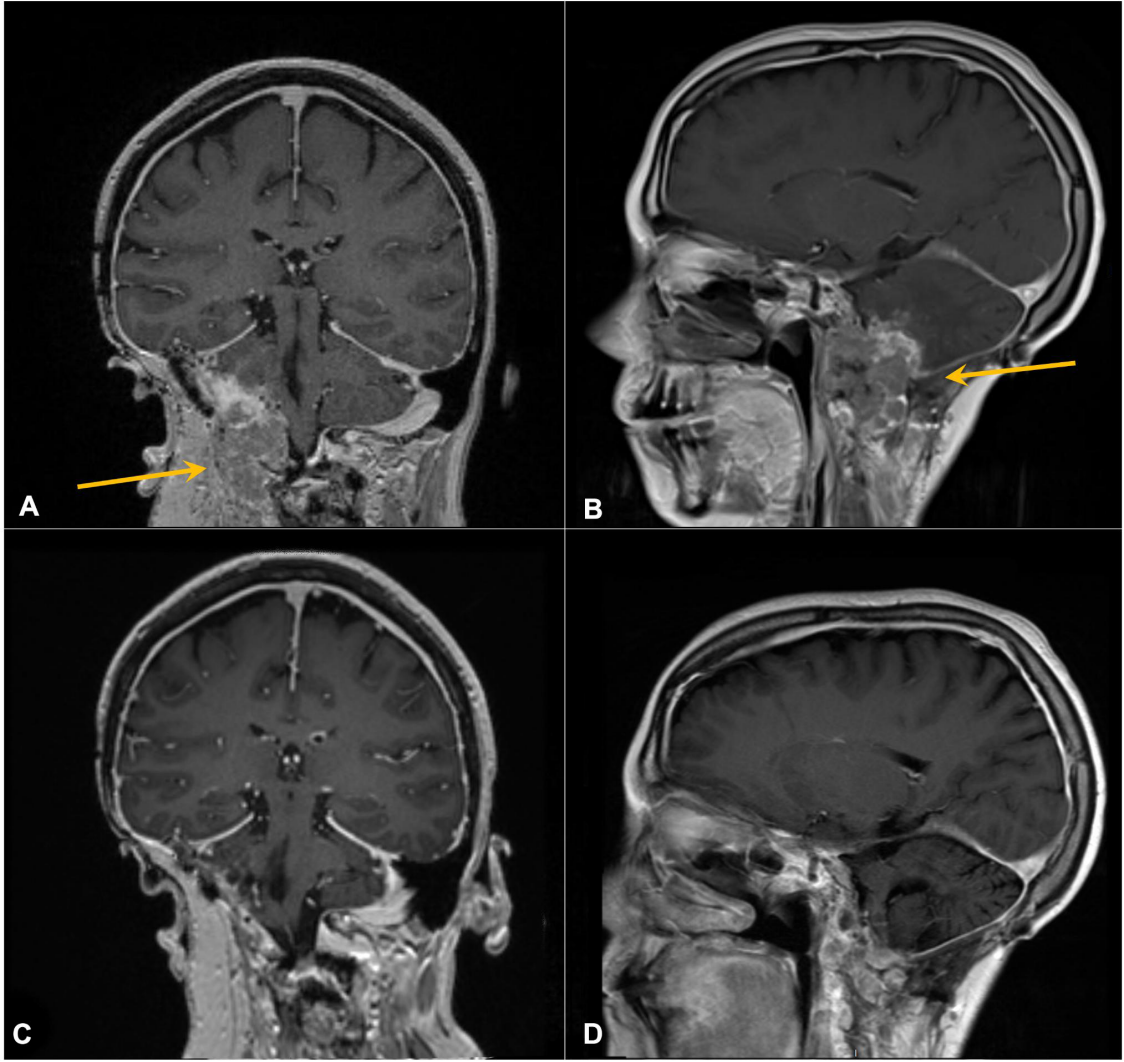
Contrast-enhanced coronal **(A, C)** and sagittal **(B, D)** T1 MRI of the brain. Arrows in panels A and B mark enhancing right cerebellar/skull base prior to pembrolizumab. **(C, D)** Following six cycles of therapy, demonstrating response.

Having the *PBRM1* mutation, a mutation possibly associated with immune therapy response in RCC, pembrolizumab was administered at standard flat dosing of 200 mg every 3 weeks starting in September 2019 (30 months from R1). The patient soon reported improvement in her symptoms and, after three cycles, returned to full-time work. She experienced no significant adverse effects from therapy. Repeat imaging demonstrated a reduction in size with near resolution of mass effect (**Figures 4A, B**), and she continued ICI therapy. At the time of manuscript writing, the patient had completed 66 cycles of pembrolizumab 200 mg every 3 weeks since R2. She continues to work and enjoys high performance status and has no evidence of disease on her most recent imaging (**Figures 4C, D**), approximately 3 years 9 months from the initiation of ICI therapy and 50 months from R2.

Other cases benefiting from ICI therapy include an advanced lung cancer patient co-diagnosed with imaging-based meningioma (lacking tissue confirmation) in which both tumors continued growing on standard chemotherapy prior to seeing a positive response to nivolumab (24). Another case with atypical meningioma (grade 2)—with mismatch repair deficiency and disease extending extra-axially from a frontal convexity tumor to involving the scalp—had prior treatment with bevacizumab, temozolomide, two radiosurgeries, and seven surgical debulking procedures, but exhibited benefit from nivolumab (25). In a 1997 study, six patients with unresectable or malignant meningiomas were treated with interferon alpha-2B, with five patients showing a positive response to treatment. Of those five patients, four experienced tumor stabilization with a range of 6 to 14 months (26, 27). Furthermore, a study published in 2022 documented a slight trend in increased PD-L1 expression correlating with better outcomes and growth stabilization in pembrolizumab-treated meningioma patients (20). Twelve patients achieved a median progression-free survival (PFS) of 7.6 months, which was a favorable comparison to previous trials that had only reported PFS of 4–26 weeks (20, 28). The findings of this study also suggested that T-cell or myeloid-cell phenotypic dynamics, as well as the level of histological aggression, may dictate whether a clinical benefit or disease response is achieved from ICI therapy (20). Although limited in scope, these cases collectively support the exploration of immunotherapy as an option for the treatment of advanced meningiomas.

## Discussion

*PBRM1* is a tumor suppressor gene that codes for BAF180, a component of the chromatin remodeling complex (29). Thus, its loss of function impacts chromatin structure and downstream transcriptional and DNA repair processes (30, 31). *In vivo* experiments have demonstrated increased tumorigenesis in mice with downregulated *PBRM1*, with the greatest difference in gene expression being seen in the chemokine/chemokine receptor interaction pathway, suggesting a possible mechanism by which *PBRM1* alters cell cycle progression and proliferation (32). Other recent studies have shown that a lack of *PBRM1* subsequently results in DNA damage and dynamic chromosome instability (33).

In patients with clear cell RCC, loss-of-function mutations in *PBRM1* are common and are associated with clinical benefit from immune checkpoint inhibitors (13, 34). Braun and colleagues reported consistent results: in 189 patients with metastatic clear cell RCC receiving nivolumab or everolimus as part of a clinical trial, 55 patients had a *PBRM1* mutation, which was associated with both clinical benefit and longer PFS in nivolumab-treated patients. There was no effect noted in those treated with everolimus only.

In contrast, in a retrospective analysis conducted at three Chinese institutions, presumably in Asian patients, *PBRM1* mutations were infrequent in patients with non-small cell lung cancer (84/2,767, 3%). This analysis demonstrated that *PBRM1* may be potentially associated with poorer survival in patients treated with immunotherapy, despite previous reports suggesting a correlation between *PBRM1* mutations and increased neoantigens (35, 36).

*PBRM1* genetic alterations are infrequent (2.8%) in meningiomas, and alterations are usually associated with high-grade meningiomas (37). Unfortunately, the genomics data we obtained from Caris Life Sciences did not contain information regarding the tumor grade of the included patients. However, in a recent case series of 850 patients with meningiomas that were grade 1 (220/850, 26%), grade 2 (441/850, 52%), and grade 3 (176/850, 20%) (13 cases were not graded due to inadequate specimens), only 16 had an inactivating mutation in *PBRM1* (1.9%) (14). The majority of the 16 *PBRM1* meningioma cases (11) had papillary histologic features that were higher grade (2/16 grade 1, 8/16 grade 2, and 6/16 grade 3), all were microsatellite stable and had a low median TMB of 2.1 Muts/Mb, and five cases had an overlap mutation with *BAP1*. Our analyses of 399 meningioma patients undergoing NGS testing demonstrated that patients with *PBRM1* alterations had likely lower overall survival. The frequency (2.5%) of *PBRM1* alterations in meningiomas in our analysis matches published literature. Despite PBRM1 mutations rarely occurring in meningioma, this represents a potential therapeutic investigation.

*BAP1* was originally identified as a *BRCA1*-interacting protein and encodes a de-ubiquitinating enzyme that is involved in many processes (38). *BAP1* can act as a subunit of the Polycomb Repressive De-ubiquitinase complex (PR-DUB), which reverses the ubiquitinating activity of Polycomb Repressive Complex 1 (PRC1); one key PR-DUB substrate is histone H2A ubiquitinated at lysine 119, so *BAP1* normally acts to modulate chromatin structure and cellular epigenetic status (39, 40). Thus, loss of *BAP1* function is thought to affect DNA repair and transcription processes that are affected by chromatin state. Mutations in *BAP1* have been reported to correlate with positive response to immunotherapy (41), perhaps by similar mechanisms as for *PBRM1*, but *BAP1* alterations are even rarer (<1%) (37).

This report details our experiences with a patient with advanced meningiomas and illustrates the challenges associated with treating these malignancies. Our patient received proton therapy and aggressive multi-team surgery, underwent a first-in-human early-phase clinical trial, and was treated with a somatostatin analog for many years. Our patient’s meningioma demonstrated stable microsatellite status and PD-L1 negativity, but with a TMB of 4 Muts/Mb, which is higher than reported for atypical meningioma (mean 1.8 Muts/Mb) but lower than TMBs in other tumors (melanomas, many lung cancers, and microsatellite instability (MSI)-high cancers) (25) for which ICIs have FDA labeling. Thus, it seems unlikely that PD-L1 or TMB levels explain the positive response to immunotherapy.

The genomics report demonstrated probable loss-of-function alterations (large deletions) in the tumor suppressor gene *PBRM1* on chromosome 3p21. Our patient had a *PBRM1* deletion involving exons 2 through 12. Missense mutations in the bromodomain regions have been shown to result in the tumor suppressor activity of *PBRM1*, especially in the bromodomain 2 (42). Further, the bromodomains have also been found to be essential in the chromatin complex interaction (42, 43). Even though it is tempting to suggest that loss of function of *PBRM1* and/or BAP1 plays a role in the positive response of our patient to pembrolizumab, the role of mutations in *PBRM1* has yet to be well-characterized. Our genomics analysis on meningioma patients demonstrated 90% (9/10) patients having *PBRM1* mutations with unknown oncogenic significance. The patient in our case report had a reported exon loss in *PBRM1*, which may result in truncating mutations leading to a loss of function as a tumor suppressor. Truncating or splice site mutations appear to be the majority of reported *PBRM1* oncogenic alterations based on the cBioPortal database (44, 45). This suggests that despite *PBRM1* being a potential biomarker for immunotherapy, heterogeneity in tumors may present challenges when validating biomarkers as a response to immunotherapy.

The current therapeutic landscape remains limited in meningioma, but treatments targeted to actionable mutations are promising. In addition to immunotherapy, ongoing clinical trials are under investigation involving FAK inhibition in patients based on preclinical synthetic lethality seen with NF2 loss and FAK inhibition (46). Despite evidence of *PBRM1* loss contributing to genomic instability or neoantigen production, a majority of the reported literature is preclinical in nature, and the concept requires further research to validate *PBRM1* as a marker for immunotherapy response. Our experience with immunotherapy in treating meningioma patients mirrors that observed in patients with other malignancies—i.e., while a substantial percentage of patients may have a positive or even exceptional response, others may not respond even though their tumors may possess a marker that would potentially predict a positive response. Our findings expand this paradigm to aggressive meningiomas from the positive outcome of our patient case, which adds to the limited previous literature demonstrating positive responses of these malignancies to immunotherapy.

## Data Availability

The original contributions presented in the study are included in the article/supplementary material. Further inquiries can be directed to the corresponding author.
